# Case Reports in Pediatric Dentistry Journals: A Systematic Review about Their Effect on Impact Factor and Future Investigations

**DOI:** 10.3390/dj7040103

**Published:** 2019-10-24

**Authors:** Romeo Patini, Edoardo Staderini, Andrea Camodeca, Federica Guglielmi, Patrizia Gallenzi

**Affiliations:** Fondazione Policlinico Universitario A. Gemelli IRCCS, Institute of Dentistry and Maxillofacial Surgery, Università Cattolica del Sacro Cuore, 00168 Rome, Italy; romeo.patini@unicatt.it (R.P.); andrecamo@tiscali.it (A.C.); fe.guglielmi@gmail.com (F.G.); patrizia.gallenzi@unicatt.it (P.G.)

**Keywords:** systematic review, pediatric dentistry, evidence-based dentistry, case reports, journal impact factor

## Abstract

*Background:* The effects of publishing case reports on journal impact factor and their impact on future research in pediatric dentistry has not been clearly evaluated yet. *Aim.* To assess the relevance and role of case reports in pediatric dentistry. *Methods:* A systematic review (PROSPERO registration number: CRD42018108621) of all case reports published between 2011 and 2012 in the three major pediatric dentistry journals was performed manually. Data regarding citations of each report were acquired from the Institute for Scientific Information database available online. The authors analyzed information regarding citations (number, percentage, and mean) received by each case report and considered their relation with the 2013 journal impact factor. *Results:* Case reports accounted for almost sixteen per cent of all articles published between 2011 and 2012. The citation rate of case reports was generally low and the highest mean citation was 0.5. This review revealed that 6 (9.52%) case reports had at least 5 citations and that the majority of the citing articles were also case reports (27.78%) or narrative reviews (25%). *Conclusions:* The publication of case reports affected the journal impact factor in a negative way, this influence is closely related to the percentage of the published case reports. Case reports about innovative topics, describing rare diseases, syndromes, and pathologies were more frequently cited.

## 1. Introduction

In medicine, a case report (CR) in scientific literature is the detailed report of the symptoms, signs, diagnosis, treatment, and follow-up of a single clinical observation [1]. CRs may describe rare diseases, unusual therapeutic approaches, or new findings related to diagnostic or treatment issues; a literature review of other reported cases can also be contained. One of the major controversial issues related to CRs, however, is their anecdotal nature that provides only small evidence. Nonetheless some authors have recognized the significant role of CRs within medical literature because they represent the only way to share new hypothesis or ideas whose value could be confirmed or rejected by higher level evidence research [2,3].

Since CRs are characterized by methodological standards that could be assessed as limitations (i.e., no statistical sampling), they are normally considered the base of the pyramid of clinical evidence [4]. However, in the last years, some authors proposed a modification to the pyramid of evidence: the pyramid should be redesigned by removing systematic reviews and using them as a lens to better analyze other types of studies. In this way, systematic reviews and meta-analysis are merely tools to validate and apply the evidence highlighted by the study with a traditionally lower level of evidence [5]. This new structure therefore gives more dignity to the level of evidence represented by CRs. Their aims, in fact, include the description of previously undetected or unknown diseases, innovative treatments, rare manifestations, and original hypotheses regarding the aetiopathogenesis of disease, recognizing adverse/beneficial effects of drugs and medications [3]. Furthermore, clinical trials usually only investigate one or few variables and do not reflect the whole picture of a multidisciplinary medical situation, on the other hand CRs often detail in depth many aspects of the patient’s medical history and current situation [6]. In order to avoid the typical sub-optimal reporting of some CRs, which hinders their use to help guide clinical practice, and with the aim of providing relevant information reporting guidelines (CARE – CAse REport - guidelines) have been developed [7]. From the development of the CARE guidelines, in fact, the CRs adhering to these guidelines are more likely to be published. Nonetheless, some journals continue to reject CRs submissions without considering that they often represent the only way to present new signs and symptoms of some rare diseases or present new diagnostic and therapeutic techniques.

The only evident reason for this rejection is the preconception that the publication of the CRs lowers the journal’s impact factor (IF).

The IF of a journal is the number of citations, received in a year, of articles published in that journal in the previous two years, divided by the total number of articles published in the same time-frame [8]. IF was developed by the Institute of Scientific Information (ISI) in the 1960s in order to assist libraries in comparing/ranking journals in a subject category [9,10], but over the years, it has evolved to become a tool to evaluate the scientific publications of researchers if not the researchers themselves. IF is directly proportional to the yearly average number of citations to recent articles published in that journal and inversely proportional to the number of published papers. Since CRs have low citation rates [2,11] they irredeemably affect journal’s IF and very often are not accepted by many prestigious journals [1]. As it concerns pediatric dentistry (PD), CRs are generally accepted in all high-impact journals if they provide relevant contribution to early diagnosis and new insights into clinical conditions.

As already reported by Nabil and Samman, there are two issues regarding CRs to be investigated. Firstly, the number of citations of CRs in high-ranking journals, as anindicator of the influence of CRs on the IF [12]. Secondly, the citation rate of CRs within PD, as an indicator of the long-term effect of CRs on future research and patient care.

The present review aims to analyze both the abovementioned issues in order to understand the significance and role of CRs in field of PD.

## 2. Materials and Methods

The Preferred Reporting Items for Systematic Review and Meta-Analyses (PRISMA) checklist was used as a guideline for conducting and reporting the present systematic review (Figure S1) [13].

### 2.1. Protocol and Registration

The protocol for this systematic review was registered on PROSPERO (CRD42018108621).

### 2.2. Selection Criteria

All CRs published in major English language PD journals (Table 1) from January 2011 to December 2012 were manually searched by the first two authors. The authors considered as “major” the PD journals with stable and continuous IF throughout the years. The period was selected in order to give enough time for acquiring citations from published articles. Potential CRs were identified through the analysis of title and abstract of all published articles in 2011 and 2012 within each journal. In the case of insufficient data to make a decision regarding the inclusion, the full text of the articles was retrieved.

Studies were included if they were case-report published in English in a major PD journal (Table 1) and conducted on human subjects.

Excluded articles included: non-English publications; reports of in vitro or animal experiments; reports of more than 5 patients; articles without complete description of demographic and clinical data of every patient; articles not citable according to the Institute for Scientific Information Web of Science (ISI WOS) and grey literature.

### 2.3. Study Selection

Two reviewers (RP and ES) screened and evaluated the titles and abstracts of the retrieved studies independently and in duplicate using the abovementioned inclusion criteria. The assessment of full-text articles was also conducted by the same reviewers. The author supervisor (PG) was involved for solving any disagreements through the discussion.

All included CRs were further divided into rare disease or pathology (RDP) or new treatment or diagnostic method (NTD).

All information regarding the citations were retrieved from ISI online databases and collected during the third week of August 2018 since the authors expected only minimal changes to the number of citations over the time during which the study was conducted. Reviewers extracted two different data regarding citations: total number of citations received in 2013 in order to give an answer to the first question of this review (the influence of CRs on journal IF) and total amount of citations received from the date the CR was citable to the date of data collection in order to answer the second question of the review (the impact of CRs on PD).

### 2.4. Data Collection Process

**Figure 1:**
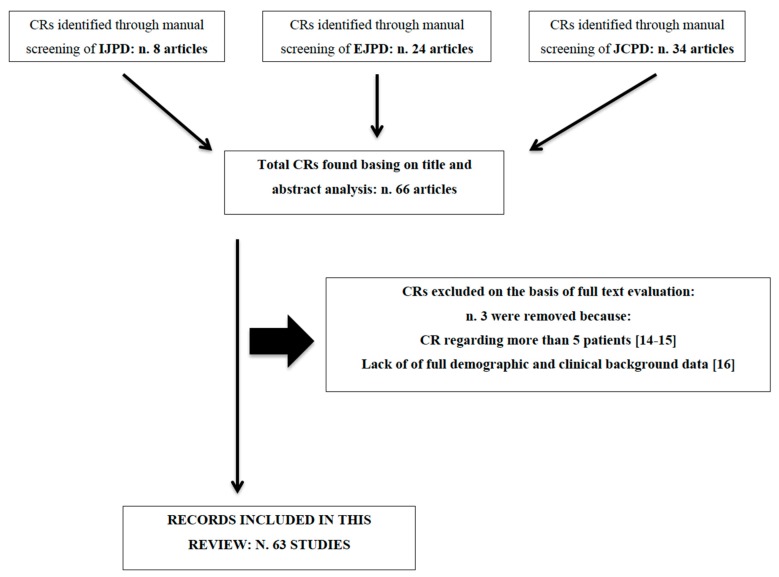
Flow chart of the search strategy. CR = Case Report; IJPD = International Journal of Paediatric Dentistry; EJPD = European Journal of Paediatric Dentistry; JCPD = Journal of Clinical Pediatric Dentistry [14,15,16].

The screening and the data collecting processes were carried on using specially designed data extraction forms. For studies apparently fitting with the inclusion criteria, or in case of title and abstract not providing sufficient information for a clear decision, the reviewers analyzed the full report. The studies excluded after full-text evaluation along with the reasons for exclusion are reported in Figure 1.

### 2.5. Outcomes

The primary outcome was the influence of publication of CRs on journal IF; in order to pursue this purpose, the authors evaluated the number/percentage of CRs present in each PD journal and their relation to the journal IF. With the aim of deeply evaluating the primary outcome for every CR published in the selected journals, the authors retrieved such elements: the total number and mean citations, the influence of the publication topic (RDP or NTD), and of the eventual inclusion of literature review on the citation rate.

The secondary outcome was the impact of the publication of CRs on future research in PD; such objective was pursued by retrieving data on the frequency of CRs citations from the day they were citable to the third week of August 2018. The authors recorded the number of citations for each included CR but, besides that, they retrieved all the non-cited CRs and all CRs with at least 5 citations along with all the citing articles. Non-cited CRs and CRs with at least 5 citations were divided according to their topic (RDP or NTD) whereas the citing articles were analyzed collecting data about the citing journals IF and the type of study (randomized controlled trial (RCT), systematic review (SR), narrative review, CR, prospective study, retrospective study, cross sectional, editorial) through analysis of their title and abstracts. In the case of insufficient data to categorize the citing articles, the full text was obtained.

## 3. Results

The final search of the ISI WOS citable CRs published from January 2011 to December 2012 in major English language PD journals retrieved 66 results, respectively: 8 published in the International Journal of Paediatric Dentistry (IJPD); 24 published by the European Journal of Paediatric Dentistry (EJPD) and 9 by the Journal of Clinical Pediatric Dentistry (JCPD). Among these results 2 CRs were excluded since they reported data regarding more than 5 patients and an additional CR was excluded because of lack of complete demographic and clinical data regarding the patient.

The flow chart of the search strategy along with references of the three excluded CRs is shown in Figure 1.

### 3.1. First Outcome: The Effect of CRs on Journal IF

In the biennium comprised between January 2011 and December 2012, the JCPD published the highest number of CRs followed by EJPD and IJPD; this order remains unchanged even considering the percentage of CRs published on the total articles (Table 2). When considering the number of citations received by CRs, a lack of correspondence between the number of CRs published and the citations received can be noted (Table 2). In fact, the IJPD, which published less than a quarter of the CRs published by JCPD, received only one citation less. While the EJPD, which published two thirds of the CRs published by JCPD received only one citation more. Analyzing the citation mean and the number of published CRs, an inversely proportional relationship between the numbers of published CRs and of citations that such CRs obtain can be noted. Surprisingly, IJPD had the highest mean (0.5) despite having published the least number of CRs and the JCPD showed opposite values, with over 30 published CRs and the lowest mean of citations (0.15). The journal IF was analyzed in light of the percentage of published CRs and an inverse proportionality was noted: IJPD had the lowest percentage of published CRs (6.35%) and the highest IF (1.54), while the JCPD showed opposite values (the highest percentage of published CRs, 22.6%, and the lowest IF, 0.31); EJPD showed intermediate values between the other two journals. In addition to these analyses the authors attempted to calculate a hypothetical IF without CRs: such result was obtained calculating the IF without considering the CRs published along with the citations they acquired. When the hypothetical and the real journal IF in 2013 were compared, the authors discovered that the two variables follow a precise pattern: the more CRs the journal publishes, the more the journal IF is dragged down (Table 2).

The authors, moreover, considered the category of CRs described and they highlighted that CRs describing RDP irrefutably have more citations in all of the analyzed journals (Table 3). Such CRs, therefore, were further analyzed to evaluate if the presence of a literature review improved the citation rate. Regarding this point, not all the results that emerged comply with each other since CRs published on IJPD seemed to retrieve more citations if they were performed without a literature review; on the contrary, CRs published on EJPD benefited from a higher number of citation if performed with a literature review. The CRs describing RDP, published on JCPD, seemed to receive the same citation mean with or without literature review (Table 4).

### 3.2. Second Outcome: The Impact of CRs on the PD Specialty and Future Research

Of all the CRs analyzed, only 6 obtained at least 5 citations considering the time-frame between the availability of the article on ISI WOS and the date of data collection (third week of August 2018). Such 6 CRs were equally distributed among the 3 journals and no proportion between this value and the number of CRs published within each journal was noted. Among the 6 highly cited CRs, there were 5 CRs describing RDP and only 1 CR describing NTD (Table 5). From this data, the author could infer that only 9.52% of CRs received at least 5 citations by August 2018, with CRs describing RDP having a strongly higher possibility (13.16%) compared to CRs describing NTD (4%). Analyzing CRs published during the biennium comprised between January 2011 and December 2012 that received no citations until the third week of August 2018, authors observed that their number amounted to 17 (26.98%). Of such CRs, 6 were published by the EJPD and 11 by the JCPD; these values show a direct proportionality to the number of CRs published within each journal. Among the 17 un-cited CRs, there were 6 CRs describing RDP (15.79%) and 11 CRs describing NTD (44%) (Table 5). As above reported, CRs describing NTD had a lower possibility of citation than CRs describing RDP. All the articles citing the CRs cited more than 5 times were analyzed and classified following the type of study and the citing journal IF (Table 6). The majority belonged to CRs (27.78%), narrative reviews (25%), and retrospective studies (13.89%). It was also highlighted that 19.45% of them had a high level of evidence: SRs (16.67%) and RCTs (2.78%). The 38.89% of citing articles were published on a journal with IF between 1.000 and 1.999; the 25% from 0 to 0.999, and the 11.11% of them on journals without IF. The remaining 25% of the CRs received citations from articles published in journals with IF over 2.000 (Table 6). The authors also investigated the topics of highly cited published CRs: 5 of them (83.33%) dealt with genetic or autoimmune diseases or syndromes.

## 4. Discussion

Evidence-based medicine (EBM) is defined as the conscientious, explicit, and judicious use of current best evidence for health care [17]. Even if CRs are commonly located at the base of the EBM pyramid because of their limitations in terms of methodology and statistical analysis [18], it has to be also considered that CRs may introduce new concepts in clinical practice when they deal with rare pathologies and syndromes; in such cases, in fact, it is virtually impossible to produce papers with a higher level of evidence. Publishing CRs, moreover, has some advantages: CRs are an optimal way to present new techniques [3], diagnostic methods [19], or to highlight unknown disease associations [20], they can provide inspiration for publishing future research with a higher strength of evidence [21] or serve as an opportunity for students for training their academic writing skills. This review has also shown that some CRs are able to obtain a fair number of citations; CRs dealing with rare diseases and patients affected by syndromes, in fact, seem to benefit from a higher rate of citations [22,23,24,25]. Despite the numerous advantages reported, scientific journals tend to publish few CRs because they are believed to be able to acquire a limited number of citations. The limited number of citations would have a negative effect on the journal IF [26]. Journal’s editors and reviewers, however, should consider that the aim of CRs is different from that of high-quality evidence ones, but not necessarily less contributing to scientific knowledge. In fact, Vandenbroucke in his editorial described the different role of CRs and higher-level evidence research: The former aims at introducing potential revisions of think tank hypotheses or ideas, while the latter has the task of confirming these new hypotheses using rigorous methodology [19]. For this reason, blocking, or greatly reducing, the publication of CRs can lead to the arrest of the catalysis process that favors the writing of papers with higher strength of evidence. Another side effect of the low CRs acceptance rate by IF-equipped journals is the authors’ forced choice to publish reports in journals providing an article processing charge (APC). Very often, such APCs are so expensive that they prevent authors from publishing if they do not have adequate funding. This editorial policy also poses ethical issues. Despite what has been previously reported, this review has shown that there is proportion between the number of CRs published and the percentage of un-cited ones. According to this data, it is reasonable to think that the greater the number of CRs published, the greater the lowering of journal’s IF. However, it is desirable that journals not only consider the editorial aspect of what they publish but also analyze the clinical growth that their articles produce for practitioners. The addition of a review of the literature background adds further value about the knowledge rare disease and it is used in order to make the publication more interesting; however, the present review also demonstrated that journals characterized by an above average IF are not influenced by the presence of a literature review in the evaluation of the quality of publications.

As it regards the biennium 2011–2012, it emerges that the percentage of CRs (16%) published in pediatric dentistry journals represents twice the percentage of CRs (7%) published in general medical journals [27]. The review demonstrated that the journal IF is not related with the percentage of published CRs. This finding shows that, probably, articles receive more citations mainly according to their methodological quality [22,28]. From this review, it emerges that the higher the percentage of CRs published, the most the journal’s IF is lowered. A previous systematic review regarding the effects of publishing CRs in oral and maxillofacial surgery on the journal IF stressed that there are several ways to manipulate journal IF, thus biasing the influence of CRs on journal IF [12]. Since IF is a ratio of the citations of every type of article published to the number of articles that are deemed citable by ISI definition, journal editors can easily control IF by publishing articles within special categories (like the ‘Letters to the Editor’, for example) that modify the numerator but not the denominator of the ratio [9,26]. Such a ploy was not found in this analysis. In the current literature, it is possible to find different categories of CRs, among which the ones that are more likely to be published include: CRs with rare diseases, novel association between two or more conditions in the same patient, and disease management in the case of unexpected evolution/complication of the condition [12,29]. In this review, the authors divided CRs into RDP and NTD in order to try to investigate if there were differences in the citation pattern of these two types of CR. CRs describing RDP are more cited overall and within each journal. This finding could be explained noting that the majority of PD publications deal with rare diseases and syndromes, which, inevitably, have to be treated in the form of CRs.

Although this review has analyzed the methodological strictness of the articles citing the CRs, it is not prudent to consider completely correct the data that show a relatively low rate of citations of CRs by SR and RCT (respectively 16.67% and 2.78%) and construe them as a lack of ability to stimulate high-level evidence because it is important to note that the rare nature of some diseases makes it impossible to reach an adequate sample for high-level evidence studies [20,21,22,23,24,25,26,27,28,29,30,31,32,33,34,35,36]. Almost all of the CRs that have been analyzed in this article, in fact, deal with RDP and such arguments, as is known, do not provide enough data to publish RCT or SR. This could be considered as a partial explanation of why CRs (especially ones dealing with RDP) do not induce higher quality evidence study. During the biennium 2011–2012, less than 10% of published CRs obtained at least five citations up to August 2018. The un-cited CRs were almost three times the highly cited (>4 citations). The percentage of highly cited and un-cited CRs were markedly different among journals: IJPD had the highest percentage of highly cited CRs and the lowest percentage of un-cited ones whereas JCPD showed an opposite trend. If it is considered that JCPD was the journal with the highest number of CRs published in the period analyzed and that IJPD was the one with less CRs published (and that no article published in IJPD remained un-cited), it is clear that editorial policies that do not exclude CRs a priori but that evaluating their methodological strictness is beneficial for the journal IF. CRs dealing with RDP had a higher citation rate and were less likely to be not cited; this finding is probably connected to the peculiarity of the pediatric dentistry discipline. The majority of the CRs published in the analyzed PD journals were cited by other CRs or by narrative reviews. This is an alarming element which shows that CRs in the PD specialty apparently trigger a cyclic citation spiral that does not add consistency to the strength of evidence (in the case of CRs) or that relies on articles with a high number of citations (in the case of narrative reviews). Based on the results of the review, there is an association between the subject and the citation rate of the CRs. Genetic or autoimmune diseases and syndromes made up over 83% of the case reports that received more than 4 citations. Understanding the impact of an article on real scientific knowledge is not easy. One of the limitations of this review is that the authors tried to do it by analyzing the citations that the article received, but this simplification may not be entirely reliable. Another very important limitation is that the purpose with which the articles received the citations was not investigated. In fact, authors could have been mentioned in an article for methodological reasons or to move some criticism to its structure or to the data analysis.

## 5. Conclusions

In conclusion, the publication of CRs remains a relevant focal point for scientific knowledge and it should be continued. Authors and editors should spend more time in carefully considering the information enclosed in the submitted CRs in order to improve its usefulness. When writing a CR on PD topics, authors should consider whether sharing their own results brings real benefits to the PD community and editors should select articles that deal with recent topics and describe RDP, since these will receive a higher number of citations and will influence future research. This review confirms that also in PD journals the publication of CR has a negative effect on their IF proportionally to the percentage of CRs published. Attempts to modify the journal IF could reduce the effect of the publication of CR in it, but this action remains doubtful and should be discussed in future publications.

## Figures and Tables

**Table 1:** Major PD journals and their 2013 impact factor.

Major Paediatric Dentistry Journals	2013 Impact Factor
International Journal of Paediatric Dentistry	1.54
European Journal of Paediatric Dentistry	0.48
Journal of Clinical Pediatric Dentistry	0.31

PD = Pediatric Dentistry.

**Table 2:** CRs in major PD journals and their impact on journal IF.

Journal	Year	CR Articles	Total Articles	CR/Total Articles	Percentage CR/Articles	Total CR Citations 2013	Total Articles Citations 2013	CR Citation/ CR Articles	Mean CR Citations	2013 IF	IF Without CR	Difference	% Difference
**IJPD**	2011	5	64	5/64	7.81%	4	116	4/5	0.8				
	2012	3	62	3/62	4.84%	0	78	0/3	0				
	Total	8	126	8/126	6.35%	4	194	4/8	0.5	194/126 = 1.54	190/118 = 1.61	0.07	4.55%
**EJPD**	2011	9	55	9/55	16.36%	5	38	5/9	0.56				
	2012	13	67	13/67	18.06%	1	21	1/13	0.08				
	Total	22	122	22/122	19.40%	6	59	6/22	0.27	59/122 = 0.48	53/100 = 0.53	0.05	10.42%
**JCPD**	2011	13	71	13/71	18.31%	2	32	2/13	0.15				
	2012	20	75	20/75	26.67%	3	13	3/20	0.15				
	Total	33	146	33/147	22.60%	5	45	5/33	0.15	45/146 = 0.31	40/113 = 0.35	0.04	12.9%

CR = Case Report; IF = Impact Factor.

**Table 3:** Major PD journals’ citations of rare disease or pathology (RDP) and new treatment or diagnostic method (NTD) CRs.

Journal	Type of CR	Number of CR Articles	2013 Cited	Total Cited	Mean 2013	Mean Total
**IJPD**	RDP	8	4	25	4/8 = 0.50	25/8 = 3.13
	NTD	0	0	0	0	0
**EJPD**	RDP	10	4	18	4/10 = 0.40	18/10 = 1.80
	NTD	12	2	19	2/12 = 0.17	19/12 = 1.58
**JCPD**	RDP	20	5	34	5/20 = 0.25	34/20 = 1.7
	NTD	13	0	12	0/13 = 0	12/13 = 0.92
**Total**	RDP	38	13	77	13/38 = 0.34	77/38 = 2.03
	NTD	25	2	31	2/25 = 0.08	31/25 = 1.24

**Table 4:** Citations of RDP CRs published on major PD journals with or without literature review.

Journal	Literature Review	Number of CR Articles	2013 Cited	Total Cited	Mean 2013	Mean Total
**IJPD**	With	1	0	2	0/1 = 0	2/1 = 2
	Without	7	4	23	4/7 = 0.57	23/7 = 3.29
**EJPD**	With	3	2	10	2/3 = 0.67	10/3 = 3.33
	Without	7	2	8	2/7 = 0.29	8/7 = 1.14
**JCPD**	With	4	1	7	1/4 = 0.25	7/4 = 1.75
	Without	16	4	27	4/16 = 0.25	27/16 = 1.69
**Total**	With	8	3	19	3/8 = 0.38	19/8 = 2.38
	Without	30	10	58	10/30 = 0.33	58/30 = 1.93

**Table 5:** Characteristics of highly cited (at least 5 citations) and un-cited CRs in major PD journals.

Journal	Type of CR	Highly Cited CR		Highly Cited CR/ Total CR in Journal	%	Non-Cited CR		Non-Cited CR/ Total CR in Journal	%
		Number of CR Articles	Total CR			Number of CR Articles	Total CR		
IJPD	RDP	2				0			
	NTD	0	2	2/8	25.00%	0	0	0	0
EJPD	RDP	1				1			
	NTD	1	2	2/22	9.09%	5	6	6/22	27.27%
JCPD	RDP	2				5			
	NTD	0	2	2/33	6.06%	6	11	11/33	33.33%
Total	RDP	5				6			
	NTD	1	6	6/63	9.52%	11	17	17/63	26.98%
	RDP	5/38 = 13.16%				6/38 = 15.79%			
	NTD	1/25 = 4.00%				11/25 = 44.0%			

**Table 6:** Characteristics of the articles citing the highly cited CRs published in major PD journals.

Journal	Type of Study (%)	Type of Study (%)	Type of Study (%)	Type of Study (%)	Type of Study (%)	Type of Study (%)	Type of Study (%)	Type of Study (%)	Citing Journal IF (%)	Citing Journal IF (%)	Citing Journal IF (%)	Citing Journal IF (%)
	Editorial	Cross sectional	CR	Retro-spective	Narrative Review	Prospective	RCT	SR	No IF	0 < IF < 0.99	1 < IF < 1.99	IF > 2
IJPD	0	0	5 (45.45)	0	5 (45.45)	0	0	1 (9.09)	0	2 (18.18)	7 (63.64)	2 (18.18)
EJPD	1 (6.67)	1 (6.67)	1 (6.67)	3 (20.00)	3 (20.00)	1 (6.67)	0	5 (33.33)	2 (13.33)	3 (20.00)	5 (33.33)	5 (33.33)
JCPD	1 (10.00)	1 (10.00)	4 (40.00)	2 (20.00)	1 (10.00)	0	1 (10.00)	0	2 (20.00)	4 (40.00)	2 (20.00)	2 (20.00)
Total	2 (5.56)	2 (5.56)	10 (27.78)	5 (13.89)	9 (25.00)	1 (2.78)	1 (2.78)	6 (16.67)	4 (11.11)	9 (25.00)	14 (38.89)	9 (25.00)

## Supplementary Materials

The following are available online at https://www.mdpi.com/2304-6767/7/4/103/s1, Figure S1: PRISMA (Preferred Reporting Items for Systematic review and Meta-Analysis) 2009 checklist.
